# The Ramazzini Institute 13-week study on glyphosate-based herbicides at human-equivalent dose in Sprague Dawley rats: study design and first in-life endpoints evaluation

**DOI:** 10.1186/s12940-018-0393-y

**Published:** 2018-05-29

**Authors:** Simona Panzacchi, Daniele Mandrioli, Fabiana Manservisi, Luciano Bua, Laura Falcioni, Marcella Spinaci, Giovanna Galeati, Giovanni Dinelli, Rossella Miglio, Alberto Mantovani, Stefano Lorenzetti, Jianzhong Hu, Jia Chen, Melissa J. Perry, Philip J. Landrigan, Fiorella Belpoggi

**Affiliations:** 1 Cesare Maltoni Cancer Research Center (CMCRC), Ramazzini Institute (RI), Via Saliceto, 3, 40010 Bentivoglio, Bologna, Italy; 2 Department of Agricultural Sciences, University of Bologna, Viale Fanin 44, 40127 Bologna, Italy; 3 Department of Veterinary Medical Sciences, University of Bologna, Via Tolara di Sopra 50, 40064 Ozzano dell’Emilia, Bologna, Italy; 4 Department of Statistical Sciences, University of Bologna, Via Belle Arti 41, 40126 Bologna, Italy; 5 Department of Food safety, Nutrition and Veterinary Public Health, Istituto Superiore di Sanità, Viale Regina Elena 299, 00161 Rome, Italy; 6 Department of Genetics and Genomic Sciences, Icahn School of Medicine at Mount Sinai, 1425 Madison Ave, New York, NY 10029 USA; 7 Department of Environmental Medicine and Public Health, Icahn School of Medicine at Mount Sinai, New York, USA; 8 Department of Environmental and Occupational Health, Milken Institute School of Public Health, The George Washington University, 950 New Hampshire Ave, Washington, DC 20052 USA; 9 Arnhold Institute for Global Health, Icahn School of Medicine at Mount Sinai, 1216 Fifth Avenue, New York, NY 10029 USA

**Keywords:** Glyphosate, Roundup, 13-week, Sprague-Dawley rat, Glyphosate based herbicides, GBH

## Abstract

**Background:**

Glyphosate-based herbicides (GBHs) are the most widely used pesticides worldwide, and glyphosate is the active ingredient of such herbicides, including the formulation known as Roundup. The massive and increasing use of GBHs results in not only the global burden of occupational exposures, but also increased exposure to the general population. The current pilot study represents the first phase of a long-term investigation of GBHs that we are conducting over the next 5 years. In this paper, we present the study design, the first evaluation of in vivo parameters and the determination of glyphosate and its major metabolite aminomethylphosphonic acid (AMPA) in urine.

**Methods:**

We exposed Sprague-Dawley (SD) rats orally via drinking water to a dose of glyphosate equivalent to the United States Acceptable Daily Intake (US ADI) of 1.75 mg/kg bw/day, defined as the chronic Reference Dose (cRfD) determined by the US EPA, starting from prenatal life, i.e. gestational day (GD) 6 of their mothers. One cohort was continuously dosed until sexual maturity (6-week cohort) and another cohort was continuously dosed until adulthood (13-week cohort). Here we present data on general toxicity and urinary concentrations of glyphosate and its major metabolite AMPA.

**Results:**

Survival, body weight, food and water consumption of the animals were not affected by the treatment with either glyphosate or Roundup. The concentration of both glyphosate and AMPA detected in the urine of SD rats treated with glyphosate were comparable to that observed in animals treated with Roundup, with an increase in relation to the duration of treatment. The majority of glyphosate was excreted unchanged. Urinary levels of the parent compound, glyphosate, were around 100-fold higher than the level of its metabolite, AMPA.

**Conclusions:**

Glyphosate concentrations in urine showed that most part of the administered dose was excreted as unchanged parent compound upon glyphosate and Roundup exposure, with an increasing pattern of glyphosate excreted in urine in relation to the duration of treatment. The adjuvants and the other substances present in Roundup did not seem to exert a major effect on the absorption and excretion of glyphosate. Our results demonstrate that urinary glyphosate is a more relevant marker of exposure than AMPA in the rodent model.

## Background

Glyphosate [IUPAC chemical name N-(phosphonomethyl)glycine] is the most widely applied pesticide worldwide and it is an active ingredient of all glyphosate-based herbicides (GBHs), including in the formulation “Roundup” [1, 2]. It is mainly marketed as a *broad*-*spectrum* systemic *herbicide* and crop desiccant [3]. The Asia-Pacific region represents the largest supplier of glyphosate active ingredient worldwide in terms of production.. In 2016, China contributed the largest share in the Asia Pacific, and is likely to remain a dominant market for years to come. The United State trails behind the Asia-Pacific market in the production of GBHs. Latin America, Middle East and Africa are expected to grow in terms of use at a significant rate during 2017–2025 [4]. Production and use of glyphosate have risen dramatically with the introduction in 1996 of genetically modified (GM) glyphosate tolerant crop varieties. In the United States (US) glyphosate is contained in over 750 products, particularly herbicides used for intensive GM crops that have built-in tolerance to glyphosate, but also in other products used in agriculture, forestry, urban, and home applications [5]. In 2015, 89% of corn, 94% of soybeans, and 89% of cotton cropped in the US were genetically modified to be glyphosate-tolerant [6]. Only a few data on the use of individual pesticides are available for certain countries in the European Union (EU), making it difficult to find out how much glyphosate is being used by farmers [7]. However, surveys in individual countries give some indication. Glyphosate is the top ranked herbicide in United Kingdom arable crop production [8]. In Denmark, glyphosate accounts for 35% of all pesticides used in agricultural production [9]. In Germany, it has been estimated that glyphosate is used on 4.3 million hectares (39%) of agricultural land each year, with nearly two thirds applied to just 3 crops - oilseed rape, winter wheat and winter barley [10]. The EU has a strict regulation regarding the planting of GM crops (Directive EU 2015/412) [11] and GBHs are mainly applied to cereals for post-harvest desiccation purposes (wheat, rye, triticale, barley and oats), oilseeds (rapeseed, mustard seed and linseed), orchards and vineyards [12].

The massive and increasing use of GBHs leads to a global burden of occupational exposures in manufacturing workers and GBH applicators (farmers), as well as increasing exposures in the general population, as demonstrated by environmental contamination from glyphosate residues found in air [13], groundwater [14, 15], drinking-water [16], crops [17, 18], food [19, 20] and animal feed [21]. Microbial biodegradation of glyphosate occurs in soil, aquatic sediment and water. The main pathway of biodegradation of glyphosate appears to be by splitting the C–N bond to produce aminomethylphosphonic acid (AMPA), the major microbial metabolite [22]. In humans, the main exposure routes to glyphosate are inhalation and dermal exposure in the occupational setting and consumption of water and food for the general population [22]. The results of oral studies with [^14^C] glyphosate in rats, rabbits and goats indicate that absorption from the gastrointestinal tract is incomplete and amounts to up to 30% of the dose [23–25]. The most relevant routes of excretion following oral administration of glyphosate [^14^C] are feces (70–80%) and urine (20–30%) [26]. In rats, after a single oral administration of [^14^C] glyphosate, almost all radioactivity was detected in urine and feces, and the radiolabeled detected chemical was present as the unchanged parent compound [27–29]. Elimination through exhaled air was very low. AMPA was the only metabolite detected, accounting for only 0.2–0.3% of the applied dose of [^14^C] glyphosate [30]. The limited data currently available on glyphosate pharmacokinetics in vertebrates are insufficient to predict transport and fate of glyphosate in different mammalian tissues, organs and fluids in the body, and to determine whether or where bioaccumulation occurs, although animal metabolism studies indicate kidney and liver as target tissues [1].

The possible effects of GBHs on human health is the topic of intense public debate, for both its potential carcinogenic and non-carcinogenic effects, including endocrine disruption, neurotoxicity, developmental and reproductive toxicity, which might occur even at doses much lower than the ones considered for risk assessment, in particular during sensitive periods of life (such as fetal development) [5, 12, 31, 32]. Glyphosate, as the pure active substance, and GBHs may not be quite the same from the toxicological standpoint. Glyphosate formulations contain a number of so-called ‘inert’ ingredients or adjuvants to facilitate the uptake by plants, most of which are patented and not publicly known (in many countries the law does not require a full disclosure of pesticide ingredients). GBHs that contain surfactants and adjuvants might act differently than glyphosate alone [33, 34]. In fact, adjuvants might potentiate the toxic effects of glyphosate [35–38].

### The Ramazzini Institute 13-week pilot study: aims and experimental design

The present pilot study is the first phase of an integrated long-term project on GBHs that we are conducting during the next 5 years [39]. The initial focus of our pilot study is to assess techniques and methods for glyphosate detection in different matrices (results presented here), then to evaluate target organ toxicity, genotoxicity and endocrine disrupting activities, together with omics and microbiome alterations (not presented here). In our pilot study, we exposed Sprague-Dawley (SD) rats to either glyphosate or Roundup, one of the most popular branded GBHs, with a dosage considered to be “safe”, the United States Acceptable Daily Intake (US ADI) of 1.75 mg/kg bw/day, defined as the chronic Reference Dose (cRfD) determined by the US EPA [40]. The design of the pilot study derives from the 13-week cohort protocol of the National Toxicology Program (NTP) guideline Modified One-Generation study (MOG) [39, 41]. It incorporates exposure during the perinatal period (i.e., gestation and lactation) and later for 13 weeks after the pups are weaned, evaluating standard sub-chronic toxicity and functional endpoints (e.g., sperm analysis, vaginal cytology, indices of puberty and sexual differentiation) to investigate possible effects on the reproductive and endocrine systems. In order to provide more information about specific modes of action, we further integrated the 13-week cohort NTP MOG design with transcriptome analyses of potential target tissues and gut microbiome evaluation at different time-points and life stages in both dams and their offspring. The whole-transcriptome analysis can provide important mechanistic information and support the pathological evaluation of target organs and hormone analysis. The gut microbiome evaluation is a novel endpoint representing the potential role of altered balance in the gut microbiota that relate to several health disorders such as metabolic diseases, hepatic, coronary and gastrointestinal diseases (e.g., inflammatory bowel disease) [32]. The experimental plan and the endpoints investigated in the study are presented in Table 1 and Table 2.

**Table 1:** Experimental plan

Breeders			Offspring								
Group	Animals		Group	Animals^a^			Treatment^b^			End of the experiment	
	Sex	No.	N.	Sex	Cohort		Compound	Dose^c^	Age at start^d^	Cohort	
					6-week (No.)	13-week (No.)				6-week (PND)	13-week (PND)
I	F	8	I	M	8	10	Control (drinking water)	0	GD 6	70^e^	120^f^
	M	8		F	8	10					
	F + M	16		M + F	16	20					
II	F	8	II	M	8	10	Glyphosate	US ADI	GD 6	70^e^	120^f^
	M	8		F	8	10					
	F + M	16		M + F	16	20					
III	F	8	III	F	8	10	Roundup	US ADI Glyphosate equivalent	GD 6	70^e^	120^f^
	M	8		M	8	10					
	F + M	16		F + M	16	20					
Total	M + F	48		M + F	48	60					

^a^No more than 2 sisters and 2 brothers per litter

^b^Test compounds are administered ad libitum in drinking water

^c^Doses are calculated considering the Glyphosate US ADI defined as the chronic Reference Dose (cRfD) determined by the US EPA (1.75 mg/kg bw/day)

^d^Solutions are admistered to dams starting from the 6th day of pregnancy

^e^Animals are treated until the landmarks of sexual development are acquired (PND 73 ± 2)

^f^Animals are treated from embryonic life (GD 6) indirectly from dams milk until PND 28 ± 2, then directly for 90 days after weaning (until PND 125 ± 2)

**Table 2:** Summary of the endpoints and relative monitoring time points evaluated in the study, in dams and offspring (6-week and 13-week cohorts)

Endpoints	Time points	Dams	Offspring 6-week cohort	Offspring 13-week cohort
Gestation length	GD0-delivery	✓	–	–
AGD and body weight in male and female pups	PND 1	–	✓	✓
Litter size	PND 1, 4, 7, 10, 13, 16, 19, 21, 25	–	✓	✓
Live-birth index	PND 1	–	✓	✓
Survival index	PND 4, 7, 10, 13, 16, 19, 21, 25	–	✓	✓
Age and body weight at BPS in male pups	PND 35	–	✓	✓
Age and body weight at VO in female pups	PND 28	–	✓	✓
First estrous in female pups	3 days after VO	–	✓	–
Estrous cycle length and percentage of days in each stage	PND 95 - PND 116	–	–	✓
Estrous cycle prior to necropsy	PND 125 ± 2	–	–	✓
Serum hormone measures	End of lactation (dams), PND 73 ± 2 and PND 125 ± 2	✓	✓	✓
Clinical biochemistry	PND 73 ± 2 and PND 125 ± 2	–	✓	✓
Urinalysis	PND 73 ± 2 and PND 125 ± 2	–	✓	✓
Glyphosate and AMPA detection in urine	End of lactation (dams), PND 73 ± 2 and PND 125 ± 2	✓	✓	✓
Sperm counts	PND 73 ± 2 and PND 125 ± 2	–	✓	✓
Daily Sperm production	PND 73 ± 2 and PND 125 ± 2	–	✓	✓
Sperm transit time through the epididymis	PND 73 ± 2 and PND 125 ± 2	–	✓	✓
Sperm morphology	PND 73 ± 2 and PND 125 ± 2	–	✓	✓
Sperm aneuploidy	PND 73 ± 2 and PND 125 ± 2	–	✓	✓
Partial histopathology (reproductive organs, brain, liver, kidney)	End of lactation (dams)	✓	–	–
Complete histopathology	PND 73 ± 2 and PND 125 ± 2	–	✓	✓
Organ weight	End of lactation (dams), PND 73 ± 2 and PND 125 ± 2	✓	✓	✓
Micronuclei test (bone marrow)	PND 73 ± 2 and PND 125 ± 2	–	✓	✓
Transcriptome on mammary glands	End of lactation (dams), PND 73 ± 2 and PND 125 ± 2	✓	✓	✓
Transcriptome on brain	PND 125 ± 2	–	–	✓
Transcriptome on liver	End of lactation (dams), PND 73 ± 2 and PND 125 ± 2	✓	✓	✓
Transcriptome on kidneys	End of lactation (dams), PND 73 ± 2 and PND 125 ± 2	✓	✓	✓
Microbiome analysis in dams	Before mating, GD 5 (before treatment), GD 13, LD 7, LD 14	✓	–	–
Microbiome analysis in offspring	PND 7, PND 14, PND 31 (before puberty), PND 57 (after puberty), PND 125 ± 2 (adulthood)	–	✓	✓

*GD* gestation day, *LD* lactation day, *PND* postnatal day, *AGD* anogenital distance, *VO* vaginal opening, *BPS* balano preputial separation

The protocol of the pilot study commences with exposure from gestation day (GD) 6 (implantation) continuously through pregnancy and lactation. To satisfy the need to consider multiple effects across multiple life stages, at weaning the offspring were assigned to two testing cohorts at *random*, so as to have minimal differences in body weight among groups (standard deviation < 10% of the average). The first cohort (6-week cohort) was continuously dosed until full sexual maturity (Post Natal Day-PND 73 ± 2), then sacrificed. The second cohort (13-week cohort) was continuously dosed until adulthood (PND 125 ± 2), then sacrificed. Both cohorts were analyzed for post-natal developmental landmarks, microbiome, target organs toxicity and clinical pathology.

The design of the pilot study has been developed by the Ramazzini Institute in collaboration with all Institutions taking part in the overall Glyphosate Study. All of the in vivo experimental phases of the study were performed at the Ramazzini Institute, while the other collaborating Institutions have independently assessed different outcomes and endpoints of interest. In this paper, we present the study design, the first evaluation of in vivo parameters and the determination of glyphosate and its major metabolite AMPA in urine.

## Methods

### Experimental model

The study was conducted following the rules established by the Italian law regulating the use and humane treatment of animals for scientific purposes [Decreto Legislativo (D.Lgs.) N. 26, 2014. Attuazione della direttiva n. 2010/63/UE in materia di protezione degli animali utilizzati a fini scientifici. - G.U. Serie Generale, n. 61 del 14 Marzo 2014]. Before starting, the protocol was examined by the Internal Ethical Committee for approval. The protocol of the experiment was also approved and formally authorized by the ad hoc commission of the Italian Ministry of Health (ministerial approval n. 710/2015-PR). The experiment was performed on both male and female SD rats, which belong to the colony used at the Cesare Maltoni Cancer Research Center laboratories of the Ramazzini Institute (CMCRC/RI) for over 40 years. An animal disease screening program enforced by the Italian Health Authority and Research Organization for Animal Health is in place and ongoing on sentinel animals belonging to the RI colony.

Female breeders SD rats were placed individually in Polycarbonate cage (42x26x18cm; Tecniplast Buguggiate, Varese, Italy) with a single unrelated male until evidence of copulation was observed. After mating, matched females were housed separately during gestation and delivery. Newborns were housed with their mothers until weaning. Weaned offspring were housed, by sex and treatment group, not more than 3 per each cage. Cages were identified by a card indicating: study protocol code, experimental and pedigree numbers, dosage group. A shallow layer of white fir wood shavings served as bedding (supplier: Giuseppe Bordignon, Treviso, Italy). Analysis of chemical characteristics (pH, ashes, dry weight, specific weight) and possible contamination (metals, aflatoxin, polychlorobiphenyls, organophosphorus and organochlorine pesticides) of the bedding was performed by CONSULAB Laboratories (Treviso, Italy). The cages were placed on racks, inside a single room prepared for the experiment at 22 °C ± 3 °C temperature and 50 ± 20% relative humidity. Daily checks on temperature and humidity were performed. The light was artificial and a light/dark cycle of 12 h was maintained.

During the experiment SD rats received ad libitum the standard “Corticella” pellet feed supplied by Laboratorio Dottori Piccioni Srl (Piccioni Laboratory, Milan, Italy). The constituents of the diet are: ground corn (23%), barley milled (15%), soybean meal extract (20.6%), wheat middling (24%), wheat bran (2%), spray dried whey (2.5%), di-calcium phosphate (2%), calcium carbonate (1.1%), chicken meal (6%), carob bean gum (3%), sodium chloride (0.5%), mixed vitamins (0.3%). Every day, the animals drank fresh municipal tap water from glass bottles ad libitum. Both feed and water were periodically analyzed to identify possible chemical or microbiological contaminants or impurities; the analyses are included in the documentation of the experiment. The pelleted feed was tested for possible glyphosate contamination in compliance with Commission Regulation (EU) No 293/2013 [maximum residue levels (MRLs) < 1 mg/kg]. Tap drinking water was tested for possible glyphosate contamination in compliance with Directive 2008/105/EC, D.Lgs. 152/2006, Directive2006/118/EC (active substances in pesticides, including their relevant metabolites, degradation and reaction products < 0.1 μg/l).

Active ingredient glyphosate (Pestanal™ analytical standard, CAS number 1071–83-6, purity > 99,5%) was supplied from Sigma-Aldrich (Milan, Italy). The commercial formulation Roundup Bioflow (containing 360 g/L of glyphosate acid in the form of 480 g/l isopropylamine salts of glyphosate (41.5%), water (42.5%) and surfactant (16%; chemical name, CAS number and/or exact percentage have been withheld as a trade secret) was supplied from a local agricultural consortium (Consorzio Agrario dell’Emilia, Bologna, Italy). The original containers/bottles of glyphosate and Roundup were stored in its original container and kept in a ventilated storage cabinet at room temperature (22 °C ± 3 °C) throughout the study. Purity data for each batch of glyphosate and Roundup were provided by the supplier. The opening and the use date of the different batches of test substances were recorded in the raw data. An aliquot of each lot of the test article is maintained in the ventilated storage cabinet, until 5 years from the end of the main experiment. The solutions of glyphosate and Roundup were prepared by the addition of appropriate volume of tap drinking water.

### Experimental plan

Each of twenty-four virgin female SD rats (17 weeks old, 270-315 g) was cohabited outbred with one breeder male rat of the same age and strain. Every day, the females were examined for presence of sperm. Gestational day (GD) 0 was defined as the one in which the sperm was found in vaginal smears. The day on which parturition was completed was designated as lactating day (LD) 0 for the dam and PND 0 for the offspring. Each dam and delivered litter was co-housed in common nesting box during the postpartum period. Following the NTP MOG design, on PND 28, thus 28 days after the last litter was delivered, the offspring were weaned and identified by ear punch according to the Jackson Laboratory system. Sequentially, they were allocated in the same treatment group of their mother in order to have 18 males (8 for the 6-week cohort and 10 for the 13-week cohort) and 18 females (8 for the 6-week cohort and 10 for the 13-week cohort) for each dose group. No more than 2 males and 2 females from the same litter were included in the same cohort/treatment group. Altogether, 108 SD rats (54 males and 54 females) were enrolled in the post-weaning treatment phase. The experimental plan of the pilot study is outlined in Table 1. A summary of the endpoints and relative monitoring time points evaluated in the pilot study, both in dams and in the offspring (6-week and 13-week cohorts) is presented in Table 2.

Two groups of SD rats were treated with either glyphosate or Roundup diluted in tap water administered ad libitum and one group received only tap water as control. Roundup was diluted in tap water in order to obtain an equivalent dose of glyphosate of 1.75 mg/kg bw/day. During gestational and lactational periods, embryos and newborns (F1) received the test compounds mainly through their dams (F0). Glyphosate and Roundup water formulations during these periods were freshly prepared on a daily base depending on individual body weight and water consumption of dams as measured at each scheduled time point (see below). After weaning, until the end of the experiment (PND 73 ± 2 or 125 ± 2), the test substances were administered in tap water to F1 animals on the basis of the average body weight and average water consumption *per* sex and *per* experimental group, as measured at each scheduled time point (see below). Males and females were considered separately because of their difference in weight gain, body weight and water consumption.

At least every week, the exposure doses were recalculated and registered. The actual levels of test compounds that reached the fetus during gestation or that were ingested postnatally by the offspring during the period of lactation were not estimated in the present study.

Animals were monitored during the entire experimental period. The following procedures were performed:

Health status control: from the start of the experiment, animals were checked three times daily, except on Sundays and non-working days, when they were only checked twice. All observed variations from normal status were recorded.

Clinical control: status, behavior and clinical observation on the experimental animals were checked before the start of the treatment, and at least every two days until the end of the experiment. Any findings listed below were then recorded: alterations of skin, hair, eyes and mucosa; modification in production of secretions or excretions and in autonomic activity; respiratory symptoms; postural changes or changes in walk; presence of tonic or clonic contractions; unusual stereotypes and behavior.

Dams’ body weights were recorded on GD 0, 3, 6 and then daily during gestation until parturition. During lactation, dams’ body weights were recorded at LD 1, 4, 7, 10, 13, 16, 19, 21 and 25 (last measurement before weaning). Pups’ body weight by sex and litter was determined on PND 1, 4, 7, 10, 13, 16, 19, 21 and 25. After weaning, the body weight was measured twice a week, until PND 73 ± 2, then weekly until PND 125 ± 2 and before terminal sacrifices; the means of individual body weights were calculated for each group and sex.

Dams’ feed and water consumption were recorded twice weekly during gestation (GD 0, 3, 6, 9, 12, 15, 18, 21), whereas during lactation were measured at LD 1, 4, 7, 10, 13, 16, 19, 21, 25 and 28.

After weaning the daily water and feed consumption *per* cage were measured twice a week, until PND 73 ± 2, then weekly until PND 125 ± 2; the means of individual consumptions were calculated for each group and sex.

The day before the terminal sacrifices, all the animals were located individually in metabolic cages and starved for around 16 h. During this time, the animals had free access to water alone or to the programmed test compound solutions. The day after, in the morning, samples of at least 5 ml of spontaneous urine from each animal were collected and put in separate labelled tubes. Urine samples for analysis of glyphosate and AMPA excretion were obtained from 3 dams/group and from 10 (5 males + 5 females) rats/group belonging to the 6-week and 13-week cohorts.

### Glyphosate and aminomethylphosphonic acid (AMPA) detection

Analyses of glyphosate and its metabolite AMPA in drinking water, feed and urine were performed by Neotron Laboratories (Modena, Italy), an officially accredited laboratory by Accredia (Lab. N. 0026) according to European regulation UNI CEI EN ISO/IEC 17025:2005. The specification and results are maintained in the experimental documentation. The analytical method is based on liquid chromatography tandem mass spectrometry (LC-MS/MS) [42–45]. The limit of quantification (LQ) for glyphosate and AMPA corresponded to 0.10 μg/l in water, 50 μg/kg in feed, and 1 μg/kg in urine.

### Statistical analysis

Summary statistics, means ± standard deviations (sd), were calculated for continuous variables. For body weight, water and feed consumption over time further analyses were performed using multilevel mixed-effect linear regression models, to control for within subject correlation across time; moreover we have considered also the litter effect during the lactation period. Analysis of variance and Dunnett’s tests (when applicable) were also performed to compare body weight gain in different periods and consumption of food and water as mean consumption in several periods.

All tests were two tailed, with alpha set at 0.05. Statistical analyses were perfomed by using STATA version10 (Stata Corporation, College StationTexas, USA).

## Results

**Fig. 1:**
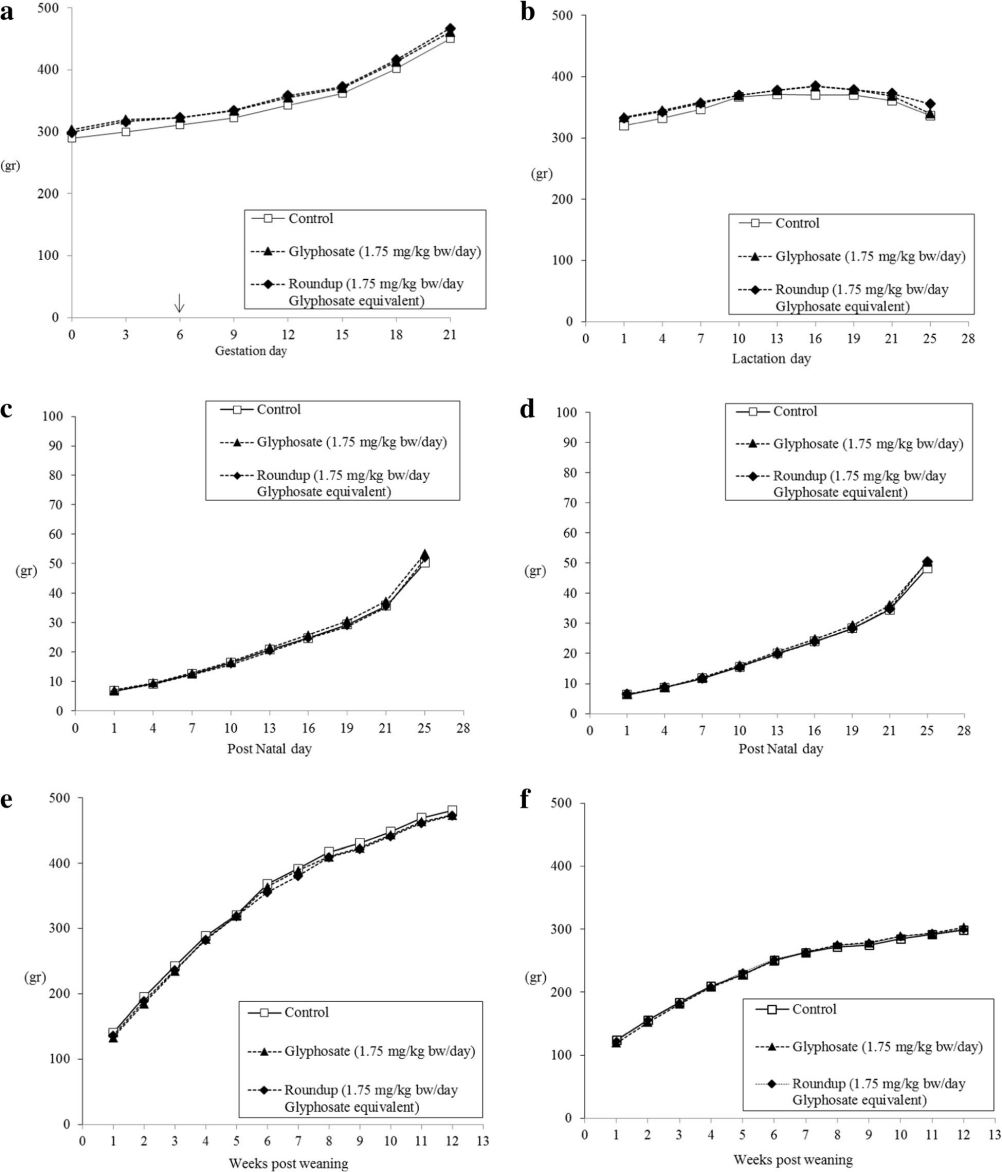
Average body weight: dams during gestation (**a**), treatment starting at gestation day 6 (↓); dams (**b**), male (**c**) and female (**d**) offspring during lactation; male (**e**) and female (**f**) offspring after weaning. At week 6 after weaning 8 male and 8 female pups per group were sacrificed

In dams, during both gestation and lactation, body weight and weight gain were not statistically different among the different groups (Fig. 1 a-b). In both female and male offspring, post weaning body weights were homogenous and no statistically significant differences in body weight gain were observed among groups (Fig. 1 c-f). All 24 dams and 108 SD rats from the 6-week (48/48) and 13-week (60/60) cohorts survived until sacrifice.

**Fig. 2:**
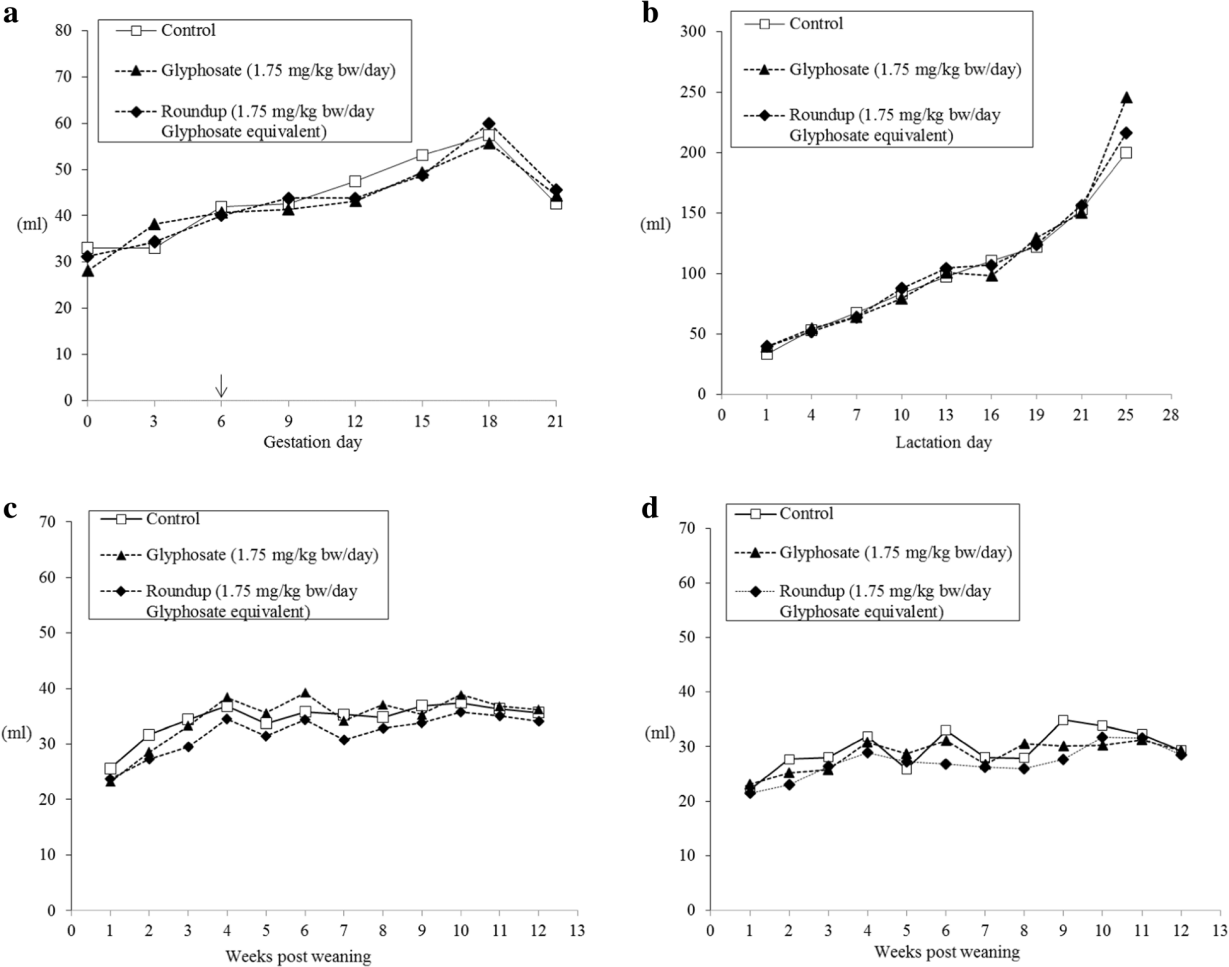
Average water consumption: dams during gestation (**a**), treatment starting at gestation day 6 (↓); dams and litter (**b**) during lactation; male (**c**) and female (**d**) offspring after weaning. At week 6 after weaning 8 male and 8 female pups per group were sacrificed

**Fig. 3:**
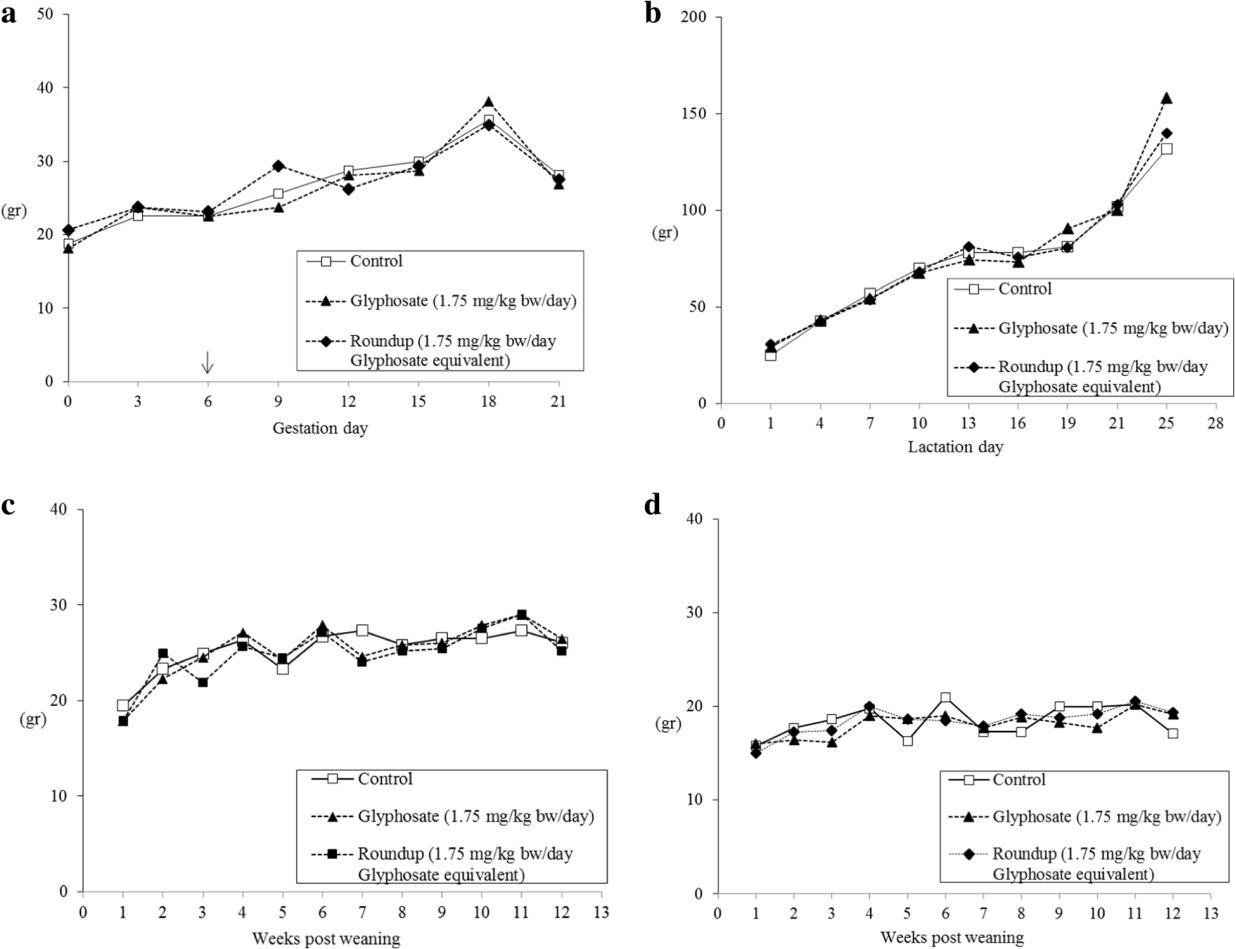
Average feed consumption: dams during gestation (**a**), treatment starting at gestation day 6 (↓); dams and litter (**b**) during lactation; male (**c**) and female (**d**) offspring after weaning. At week 6 after weaning 8 male and 8 female pups per group were sacrificed

Water and feed consumption during gestation and lactation were no different across the groups (Fig. 2 a-b and Fig. 3 a-b). Litter sizes were fully comparable among groups, with mean number of live pups: control group 13.6 (range 10–16); glyphosate group 13.3 (range 11–17); Roundup group 13.9 (range 11–16). Post weaning water and feed consumption were not affected by the treatment (Fig. 2 c-d and Fig. 3 c-d).

No unexpected clinical signs or symptoms were observed in the experimental animals during the in vivo phase. In particular, there was no clinical evidence of alterations in activity or behavior, reflexes, the eye or skin, or the respiratory, gastrointestinal, genito-urinary and cardiovascular systems.

**Fig. 4:**
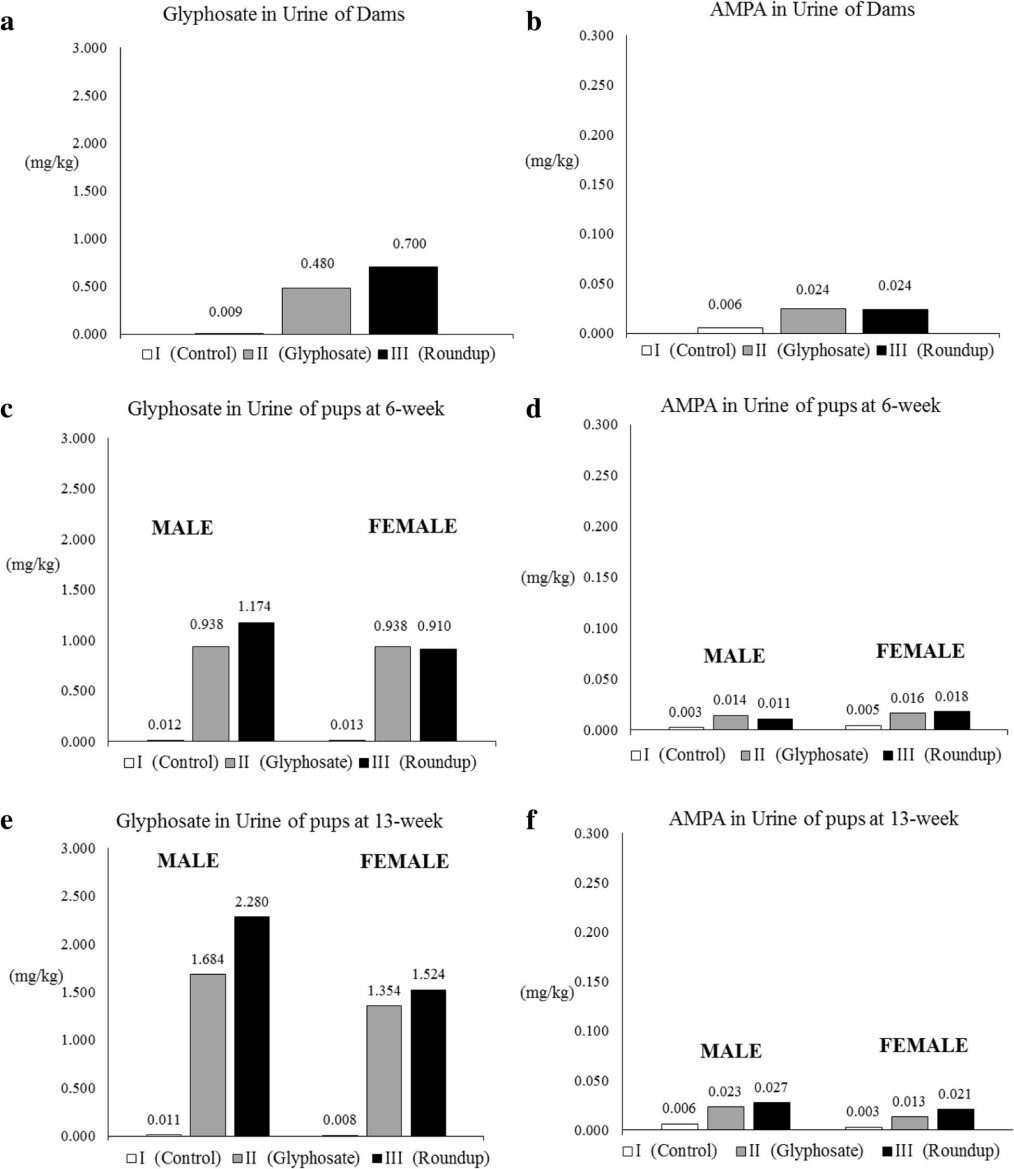
Average urinary concentrations of glyphosate and AMPA, expressed in mg/kg, collected at terminal sacrifices. Dams glyphosate (**a**) and AMPA (**b**) excretion; 6-week cohort male and female offspring; glyphosate (**c**) and AMPA (**d**) excretion; 13-week cohort male and female pups Glyphosate (**e**) and AMPA (**f**) excretion

The results of glyphosate and AMPA urinary concentrations are reported in Table 3 and Fig. 4. The urinary concentration of both glyphosate and AMPA of SD rats treated with 1.75 mg/kg bw/day of glyphosate were comparable to the ones observed in SD rats treated with Roundup dose equivalent to 1.75 mg/kg bw/day, despite limited sample size and the large standard deviations. In the control group, as expected, the glyphosate and AMPA urinary levels were all below or close to the limit of quantitation (0.001 mg/kg). In the treated SD rats, the majority of glyphosate was excreted unchanged (as parent compound), with urinary levels about 100-fold higher than that of its metabolite AMPA. For example, glyphosate and Roundup treated females in the 13-week cohort presented mean urinary levels of glyphosate respectively of 1.354 mg/kg and 1.524 mg/kg, while the AMPA levels were respectively 0.013 mg/kg and 0.021 mg/kg. In glyphosate and Roundup treated SD rats, a time-dependent increase in the mean urinary concentration of glyphosate was observed. In glyphosate and Roundup treated males, an approximate 2-fold increase of mean urinary concentration of glyphosate in the 13-week cohort (animals exposed prenatally until 125 ± 2 days after birth) compared to the 6-week cohort (animals exposed prenatally until 73 ± 2 days after birth) was observed. In glyphosate treated females, the 6-week cohort (animals exposed prenatally until 73 ± 2 days after birth) showed a 2-fold higher value of mean urinary concentration of glyphosate than the dams after weaning (exposed for 49 ± 2 days), while the 13-week cohort (animals exposed prenatally and 125 ± 2 days after birth) showed a 1.5-fold increase compared to the 6-week cohort. In the Roundup treatment group, the increase was less steep, but the time-dependent pattern was still evident. In glyphosate and Roundup treated SD rats, the levels of AMPA were comparable at the different time points in both males and females. In these animals, large standard deviations of the values of AMPA concentrations in urine have been observed, in particular for values close to the limit of quantitation as in the control groups.

**Table 3:** Glyphosate and AMPA concentration in urine. Results are reported as mean ± standard deviations

		Dams		Offspring (6-week cohort)		Offspring (13-week cohort)	
	Treatment	Glyphosate	AMPA	Glyphosate	AMPA	Glyphosate	AMPA
		(mg/kg)	(mg/kg)	(mg/kg)	(mg/kg)	(mg/kg)	(mg/kg)
Male	Control			0.012 ± 0.010	0.003 ± 0.003	0.011 ± 0.010	0.006 ± 0.004
	Glyphosate	_	_	0.938 ± 0.414	0.014 ± 0.007	1.684 ± 0.768	0.023 ± 0.012
	Roundup			1.174 ± 0.439	0.011 ± 0.005	2.280 ± 1.520	0.027 ± 0.016
Female	Control	0.009 ± 0.001	0.006 ± 0.002	0.013 ± 0.007	0.005 ± 0.001	0.008 ± 0.005	0.003 ± 0.005
	Glyphosate	0.480 ± 0.010	0.024 ± 0.002	0.938 ± 0.377	0.016 ± 0.010	1.354 ± 0.359	0.013 ± 0.006
	Roundup	0.700 ± 0.106	0.024 ± 0.001	0.910 ± 0.383	0.018 ± 0.007	1.524 ± 0.585	0.021 ± 0.007

## Discussion

Survival, body weights, food and water consumption of SD rats were not affected by the treatment with glyphosate and Roundup. Clinical changes in the animals were not observed in the various groups. Overall, both glyphosate and Roundup treatments seemed to be well tolerated, which is consistent with previous experiments performed by the US NTP [26].

Glyphosate and Roundup exposure led to comparable concentrations of glyphosate and AMPA in urine, indicating that systemic exposure does occur at the selected exposure level of 1.75 mg//kg bw/day, corresponding to the US ADI. The bioavailability of glyphosate in our study is also supported by the evident increase of glyphosate concentration in urine in relation to the length of treatment. The adjuvants and the other substances present in Roundup did not seem to exert a major effect on the absorption and excretion of glyphosate, even though mean values of glyphosate seem to be somewhat higher in the Roundup treated group. The levels in urine were also comparable between the two sexes; however, a consistent inter-individual variability was observed. In rats, glyphosate in urine appears to be the most accurate biomarker of exposure to GBHs. In fact, our results confirm previous evidence that in rodents most of the administered dose of glyphosate (98%) is excreted as unchanged parent compound, whereas the metabolite AMPA in urine is at around 0.2–0.3% of the administered dose [46]. Furthermore, with the level of exposure to glyphosate used in this pilot study, AMPA urinary values of treated animals (0.011–0.027 mg/kg) were already close to the chromatographic LQ (0.001 mg/kg) and this might limit the reliability of the measures. On the other hand, glyphosate concentration in urine of treated animals (0.480–2.280 mg/kg) resulted up to 100-fold higher than the AMPA concentration and at least 500-fold higher than the chromatographic LQ (0.001 mg/kg). Therefore, in order to assess exposure to glyphosate in rats, in particular at doses that are equal or lower than the one used in this pilot study (1.75 mg/kg bw/day), glyphosate appears to be the biomarker of choice.

The presence of negligible levels of glyphosate (0.003–0.013 mg/kg), close to the chromatographic LQ (0.001 mg/kg), in some of the urine of the control groups might reflect an ubiquitous environmental contamination at ultra-low doses of glyphosate, which is consistent with previous reports from other authors [21]. As the current limit of quantitation of glyphosate in HPLC for pelleted animal feed is 0.050 mg/kg, this represents a technical limiting factor for testing ultra-low doses of glyphosate. As reported by a recent inter laboratory comparative study on the quantitative determination of glyphosate at low levels, caution should be taken when interpreting results if the tested doses of glyphosate are close to the LQ of HPLC [47].

It is noteworthy that the commercial formulation used in this study, Roundup Bioflow, was the representative formulated product recently evaluated for the renewal of the approval of glyphosate in EU and considered in the European Food Safety Authority peer review (MON 52276) [48].

Our results seem particularly relevant in light of the massive global burden of exposure to glyphosate, as shown by the exponential increase in the last 20 years of the levels of glyphosate and AMPA measured in the urine of the general population in Germany [49] and in the US [50].

## Conclusion

We performed a pilot study on the health effects of glyphosate and its formulation Roundup administered at currently admitted doses (US ADI = 1.75 mg/kg bw/day) to SD rats. In this paper, we described the study design, the first evaluation of in vivo parameters and the determination of glyphosate and its major metabolite AMPA in urine. The treatment with either glyphosate or Roundup seemed to be overall well tolerated, consistently with previous experiments performed by the US NTP [26]. Both glyphosate and Roundup exposure led to comparable urinary concentrations of glyphosate and AMPA with an increasing pattern of glyphosate excreted in urine in relation to the duration of treatment, indicating the systemic bioavailability of the active substance and a possible mechanism of bioaccumulaton. The adjuvants and the other substances present in Roundup did not seem to exert a major effect on the absorption and excretion of glyphosate. Our results confirm that, in rodents, glyphosate in urine is the much more relevant marker of exposure than AMPA in particular at doses that are equal or lower than the one used in this pilot study (1.75 mg/kg bw/day). The evaluation of different outcomes and endpoints of interest (i.e., pathology of target organs, molecular toxicity, genotoxicity, endocrine disrupting activities, microbiome, developmental toxicity, etc.) is currently ongoing in the different partner laboratories of the project.

## Abbreviations

AMPA: Aminomethylphosphonic acid
CMCRC: Cesare Maltoni Cancer Research Center
EU: European Union
GBH: Glyphosate-based herbicides
GD: Gestational day
GM: Genetically modified
LC-MS/MS: Liquid chromatography tandem mass spectrometry
LD: Lactating day
LQ: Limit of Quantification
MOG: Modified One-Generation study
NTP: National Toxicology Program
PND: Post Natal Day
RI: Ramazzini Institute
SD: Sprague-Dawley
US ADI: United States Acceptable Daily Intake
